# Rotor–Body Echo Separation Using a Cyclic-Power-Guided Soft Mask from UAV Radar Signals

**DOI:** 10.3390/s26041382

**Published:** 2026-02-22

**Authors:** Ji’er Wang, Jing Sheng, He Tian, Bo Li

**Affiliations:** National Key Laboratory of Scattering and Radiation, Beijing 100854, China

**Keywords:** cyclic power spectrum, cyclostationary analysis, soft masking, micro-Doppler, rotor–body separation

## Abstract

Rotor-induced micro-Doppler signatures are essential for radar-based characterization of rotary-wing UAVs, but practical echoes are often dominated by a strong quasi-static body return concentrated near zero Doppler. In hovering or low-speed scenarios, rotor-induced components may intermittently overlap this near-zero region, where hard DC suppression discards informative rotor content and fragments micro-Doppler structures. Data-driven decompositions such as EMD and VMD avoid fixed cutoffs, yet without explicit constraints on rotor periodicity they are vulnerable to mode mixing and residual leakage under low-SNR conditions. This paper proposes a Cyclic-Power-Guided Soft Mask (CPGSM) framework that exploits cyclostationary periodicity as a physically grounded prior for rotor–body separation. A CPS-guided soft masking procedure consisting of a DC-dominant overlap band is first identified from quasi-static dominance; within this band, cyclic power spectrum analysis yields a continuous rotor-consistency score that guides smooth time–frequency soft allocation, while deterministic assignment is applied elsewhere. Simulations demonstrate improved micro-Doppler continuity, reduced body leakage, and more stable performance from 5–30 dB SNR compared with hard DC isolation and EMD/VMD, together with consistent rotor-speed estimates across sensing configurations.

## 1. Introduction

The proliferation of unmanned aerial vehicles (UAVs) in low-altitude airspace has increased the demand for robust radar-based detection and classification [1,2,3,4]. Radar is attractive due to its all-weather capability and its ability to capture rotor-induced micro-Doppler signatures that encode blade kinematics [5,6]. In hovering or low-speed loitering, however, the UAV body typically generates a much stronger quasi-static return concentrated near zero Doppler, which obscures weak rotor modulations in the time–frequency (TF) domain. This work targets the common regime where rotor micro-Doppler energy overlaps with the body-dominant near-zero Doppler region, making direct TF separation unreliable.

Conventional DC suppression reduces body dominance but also removes signal content around the zero Doppler crossing, fragmenting micro-Doppler structures. Decomposition methods such as empirical mode decomposition (EMD) and variational mode decomposition (VMD) provide alternatives without fixed cutoffs [7,8], yet they are not explicitly constrained by rotor periodicity; under low SNR, noise-induced mode mixing and residual leakage may yield unstable or physically ambiguous allocation [9].

In addition to decomposition-based approaches, time–frequency masking has been widely adopted in blind source separation and signal enhancement. Binary masking assigns each TF bin to a dominant component under a disjointness assumption, whereas soft weighting strategies perform continuous energy redistribution to mitigate artifacts in overlapping regions [10,11]. Recent studies have further extended soft TF masking to harmonic-aware and phase-constrained designs for micro-Doppler enhancement and radar signal separation, incorporating structural continuity or prior constraints into the weighting process [12]. These developments highlight the effectiveness of soft allocation when components exhibit TF sparsity or harmonic structure. However, most existing masking strategies remain primarily driven by instantaneous TF statistics and may become unstable when strong spectral overlap occurs in critical frequency regions such as the zero Doppler band considered in this work.

Unlike data-driven neural-network-based approaches that rely on supervised training and large annotated datasets, the separation problem considered here is addressed from a physics-guided and parameter-light perspective, focusing on transparent rotor–body separation under limited prior information and varying radar conditions [13,14,15].

Although the discussion is framed in the context of UAV sensing, the considered echo model and separation problem are equally applicable to other rotary-wing platforms, such as helicopters, where strong body returns and rotor-induced micro-Doppler coexist.

Rotor echoes are inherently periodic due to blade rotation, which induces periodic second-order statistics in the radar return and naturally motivates cyclostationary analysis [16]. The cyclic power spectrum (CPS) characterizes this spectral redundancy by revealing energy components at nonzero cyclic frequencies associated with periodic modulation. As a result, rotor-related cues can remain identifiable even when instantaneous time–frequency magnitude becomes ambiguous in the vicinity of zero Doppler. In contrast, quasi-static body returns mainly contribute to the stationary (α=0) component and do not generate pronounced cyclic-frequency signatures. This distinction motivates the use of CPS as a physically grounded prior in the proposed separation framework. However, the explicit use of CPS to guide high-fidelity rotor–body separation within the DC-overlap region has not yet been systematically explored [17,18].

We propose a Cyclic-Power-Guided Soft Mask (CPGSM) framework that formulates separation as TF-domain soft allocation restricted to a localized DC-dominant overlap band. CPS-derived cyclic-frequency evidence is mapped back to the TF plane to quantify rotor consistency and to guide energy allocation so that weak rotor structures are preserved near zero Doppler. The proposed framework is applicable to monostatic radar systems that provide time–frequency representations of slow-time echoes, including pulsed and CW/FMCW radars, provided that the sampling rate and coherent processing interval are sufficient to resolve rotor-induced periodic modulation.

The main contributions are:

1. CPS-guided rotor consistency scoring within the DC-overlap region, which maps dominant cyclic-frequency evidence to a quantitative TF-domain measure for rotor-likeness.
2. A soft mask design that smoothly bridges body- and rotor-dominant regions, enabling improved continuity of micro-Doppler structures compared with hard DC partitioning.
3. A comprehensive evaluation under severe DC overlap and degrading SNR, demonstrating consistent rotor parameter estimation and TF structure similarity across varying conditions.

These contributions are systematically validated in Section 4 through time–frequency structure similarity and rotor parameter estimation consistency, where rotor-speed estimation is used as an indicator of preserved rotational periodicity rather than as an end objective. These evaluation criteria correspond to structure preservation, consistency scoring, and robustness under DC overlap and noise, respectively.

## 2. Signal Model and Problem Statement

### 2.1. Composite Radar Echo Model of UAV Targets

We consider a monostatic radar observing a rotary-wing platform in hovering conditions. Over a short analysis interval, the received complex baseband echo is modeled as the superposition of a body component, a rotor-induced micro-Doppler component, and additive noise:

(1) $$x\left(t\right)=x_{b}\left(t\right)+x_{r}\left(t\right)+n\left(t\right)$$

Here $x_{b}\left(t\right)$ denotes the body-related echo, $x_{r}\left(t\right)$ represents the rotor-induced echo, and n(t) accounts for measurement noise and unmodeled interference.

In hovering scenarios, the body exhibits negligible radial motion within the observation window, so $x_{b}\left(t\right)$ is dominated by quasi-static scattering:

(2) $$x_{b}\left(t\right)=A_{b}\left(t\right)e^{j\phi_{b}\left(t\right)}$$

where $A_{b}\left(t\right)$ is a slowly varying amplitude term and $\phi_{b}\left(t\right)$ represents residual phase variations caused by minor vibrations, platform instability, or system imperfections. Consequently, $x_{b}\left(t\right)$ concentrates near zero Doppler in TF representations while allowing limited spectral spread caused by finite observation length and windowing.

The rotor-induced echo originates from periodically rotating blades and is the primary source of micro-Doppler modulation. Using a discrete-scatterer representation, the rotor component is written as [19,20]:

(3) $$x_{r}\left(t\right)=\sum_{k=1}^{K}A_{k}\left(t\right)exp\left\{-j\frac{4\pi }{\lambda }\left[R_{0}+r_{k}\left(t\right)\right]\right\}$$

where *K* is the number of effective rotor-related scattering contributions, $A_{k}\left(t\right)$ is a slowly varying amplitude, λ is the radar wavelength, $R_{0}$ is the nominal range, and $r_{k}\left(t\right)$ is periodic with a fundamental frequency determined by the rotor speed, leading to a time-varying phase and characteristic micro-Doppler patterns. For completeness, the instantaneous Doppler frequency follows from the phase derivative:

(4) $$f_{d,k}\left(t\right)=\frac{1}{2\pi }\frac{d}{dt}\left(\frac{4\pi }{\lambda }r_{k}\left(t\right)\right)$$

This model emphasizes the separation challenge addressed in this work: $x_{b}\left(t\right)$ is quasi-static and concentrated around zero Doppler, whereas $x_{r}\left(t\right)$ is periodic and harmonic-rich, yet its low-frequency micro-Doppler content can partially overlap with the body return under low rotor speed or limited TF resolution. The Section 2.2 analyzes the resulting TF overlap mechanism around zero Doppler and its implication for conventional separation strategies.

### 2.2. Time–Frequency Overlap Mechanism Around Zero Doppler

In this paper, the quasi-static body return is referred to as the near-zero Doppler component, whose energy is concentrated within a narrow frequency neighborhood around zero Doppler. This neighborhood is hereafter termed the DC-dominant overlap region and denoted as $F_{DC}$.

Although the body- and rotor-related echoes are physically distinct, they are often inseparable in the time–frequency (TF) domain because both contribute energy in the near-zero Doppler region. This overlap is primarily a consequence of finite-window TF analysis and limited Doppler separation under low rotor speed [21,22].

Let $X_{b}\left(t,f\right)$ denote a TF representation of x(t) obtained by STFT with an analysis window ω(τ) of duration $T_{\omega }$. For the quasi-static body echo $x_{b}\left(t\right)$, an ideal spectrum would concentrate at zero Doppler. With finite windowing, however, the body contribution is broadened by the window spectrum and can be approximated as:

(5) $$X_{b}\left(t,f\right)\approx A_{b}\left(t\right)W\left(f\right)$$

Here W(·) is the Fourier transform of ω(τ). Hence, body energy occupies a finite Doppler band around zero rather than an ideal line. The same window-induced smoothing also acts on the rotor component: rotor micro-Doppler harmonics are not observed as infinitely thin ridges in practice, and low-order components may leak into the near-zero Doppler neighborhood [23].

The overlap becomes more severe when the rotor rotation frequency is low. In this case, the lowest-order rotor harmonics lie close to zero Doppler, and the separation between rotor-induced components and the broadened body spectrum can be comparable to (or smaller than) the TF resolution determined by $T_{\omega }$. Consequently, within a small neighborhood around zero Doppler, corresponding to the DC-dominant overlap region $F_{DC}$ around zero Doppler, the observed TF coefficients contain a superposition of both components:

(6) $$X\left(t,f\right)=X_{b}\left(t,f\right)+X_{r}\left(t,f\right),\;\;\;\;\;\;f\in F_{DC}$$

This explains why hard partitioning strategies that assign all TF coefficients inside $F_{0}$ to the body inevitably discard rotor information: the near-zero Doppler band is not purely body-dominant under finite TF resolution and low rotor speed. Therefore, reliable mitigation of DC overlap requires cues beyond instantaneous TF magnitude. In particular, while $x_{b}\left(t\right)$ and $x_{r}\left(t\right)$ may overlap in Doppler, they differ in temporal statistics—the rotor-induced micro-Doppler component exhibits periodicity tied to blade rotation, whereas the body return remains quasistationary—which motivates the cyclostationary prior introduced next.

### 2.3. Cyclostationary Prior Induced by Rotor Periodicity

A key distinction between the body-related and rotor-induced echoes lies in their second-order temporal statistics. Over the analysis interval, the body return is quasistationary, whereas rotor-induced micro-Doppler exhibits periodic statistical structure due to blade rotation. This motivates cyclostationary analysis as a physically grounded prior for rotor–body separation.

A complex-valued signal x(t) is cyclostationary if its second-order statistics vary periodically with time. The cyclic autocorrelation function is defined as:

(7) $$R_{x}^{\alpha }\left(\tau \right)=\lim_{T\to \infty }\frac{1}{T}\int_{-T/2}^{T/2}E\left\{X\left(T+\frac{\tau }{2}\right)x^{*}\left(t-\frac{\tau }{2}\right)\right\}e^{-j2\pi \alpha t}dt$$

where α denotes the cyclic frequency. For a stationary process, $R_{x}^{\alpha }\left(\tau \right)$ is nonzero only at α=0; in contrast, cyclostationary signals yield nonzero components at characteristic nonzero α.

The cyclic power spectrum (CPS), obtained as the Fourier transform of the cyclic autocorrelation function $R_{x}^{\alpha }\left(\tau \right)$ with respect to τ, provides a frequency-domain description of periodic second-order statistics:

(8) $$S_{x}^{\alpha }\left(f\right)=\int_{-\infty }^{\infty }R_{x}^{\alpha }\left(\tau \right)\;e^{-j2\pi f\tau }\;d\tau .$$

Equivalently, the CPS can be interpreted in terms of spectral correlation as

(9) $$S_{x}\left(f,\alpha \right)=\lim_{T\to \infty }\frac{1}{T}\;E\left\{X_{T}\;\left(f+\frac{\alpha }{2}\right)\;X_{T}^{*}\;\left(f-\frac{\alpha }{2}\right)\right\},$$

where α denotes the cyclic frequency associated with periodic modulation. Nonzero α components indicate the presence of cyclostationary behavior, which is characteristic of rotor-induced echoes.

For rotor-induced micro-Doppler, blade periodicity gives rise to pronounced CPS components at cyclic frequencies related to the rotor rotation rate and its harmonics. These components remain identifiable even under strong body interference and low SNR, which makes cyclostationary analysis particularly attractive for rotor characterization. Such robustness has been demonstrated in prior studies, where cyclostationary phase analysis (CPA) was used to estimate micro-Doppler parameters and to distinguish small UAVs from clutter under adverse conditions [24]. By contrast, quasistationary body echoes mainly contribute to the stationary (α=0) component and do not produce prominent peaks at nonzero cyclic frequencies.

**Figure 1:**
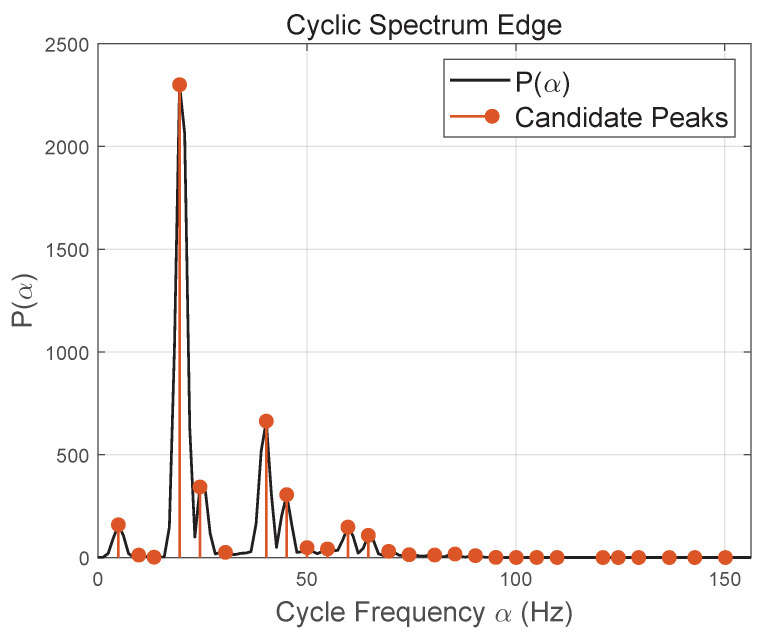
Cyclic power spectrum and modulation prior.

Beyond mere feature extraction, the cyclostationary property serves as a physically interpretable prior for rotor–body separation [25,26]. As illustrated in Figure 1, the cyclic power spectrum (CPS) exhibits distinct spectral peaks at nonzero cyclic frequencies, which correspond to periodic modulation induced by blade rotation, while the dominant component at α=0 reflects quasi-static body scattering. These nonzero cyclic-frequency peaks represent candidate rotor periodicities and provide stable evidence of rotational motion that is largely insensitive to instantaneous TF overlap and noise fluctuations. In the proposed framework, such CPS peaks are not treated as isolated features, but are subsequently exploited as a global periodicity prior to guide energy allocation and soft masking in the TF domain.

## 3. Proposed Method

### 3.1. Overall CPS-Guided Soft Masking Framework

**Figure 2:**
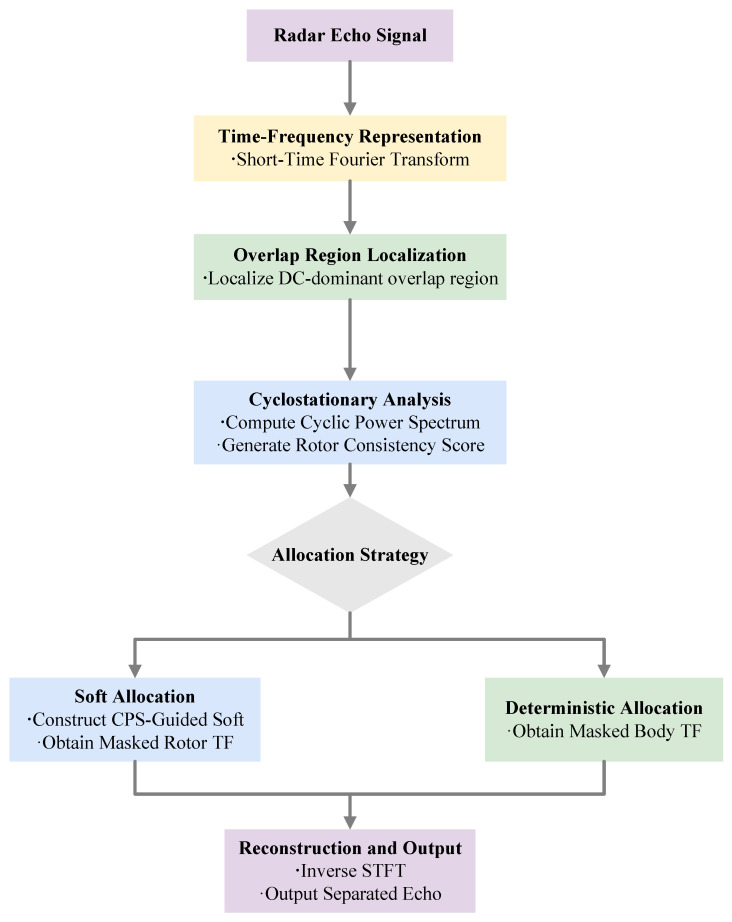
Overview of the proposed CPS-prior-guided soft mask separation pipeline.

Figure 2 summarizes the proposed CPS-prior-guided soft mask separation pipeline. The received echo is first mapped to the time–frequency (TF) domain via STFT. Since separation ambiguity primarily occurs near zero Doppler where quasi-static body scattering overlaps with low-order rotor-induced micro-Doppler components, the framework starts by localizing a DC-dominant overlap band $F_{DC}$ (Section 3.2). Within $F_{DC}$, CPS analysis is used to extract rotor periodicity and to derive a continuous rotor-consistency score (Section 3.3). This score is then mapped back to the TF plane to construct a CPS-guided soft mask for energy allocation inside $F_{DC}$ while deterministic assignment is applied outside this band (Section 3.4). Finally, the separated TF components are inverted by ISTFT to obtain the body and rotor echoes.

Overall, the method follows a CPS-guided soft masking procedure, where cyclostationary information serves as a physically grounded prior for TF-domain separation.

### 3.2. Localization of the DC-Dominant Overlap Region

As discussed in Section 2, separation ambiguity mainly concentrates near zero Doppler, where the quasi-static body return overlaps with low-order rotor-induced micro-Doppler components under finite TF resolution. The proposed method therefore first localizes a DC-dominant overlap band $F_{DC}$ and confines subsequent soft allocation to this band.

Let X(t,f) denote the complex STFT of x(t) and let ${\left|X\left(t,f\right)\right|}^{2}$ be the corresponding TF power. To characterize temporal modulation at each Doppler frequency bin *f*, we compute a cadence-domain representation by taking the discrete Fourier transform of the frame-wise TF power sequence:

(10) $$C\left(f,v\right)=|\sum_{t}{\left|X\left(t,f\right)\right|}^{2}e^{-j2\pi vt}|$$

where *v* denotes the cadence frequency. For body-dominated (quasi-static) returns, cadence energy concentrates near v≈0; rotor-induced modulation, in contrast, produces energy at nonzero cadence frequencies associated with blade periodicity. To quantify the degree of quasi-static dominance at each Doppler bin, a DC concentration index is defined based on the cadence-domain representation. Specifically, for each Doppler frequency *f*, the index is computed as

(11) $$D\left(f\right)=\sum_{v\in V_{0}}C\left(f,v\right),$$

where C(f,v) denotes the cadence-domain energy and $V_{0}$ represents a narrow low-cadence neighborhood around v=0. This concentration index measures the amount of slowly varying (quasi-static) energy at Doppler frequency *f*, with larger values indicating stronger DC dominance.

**Figure 3:**
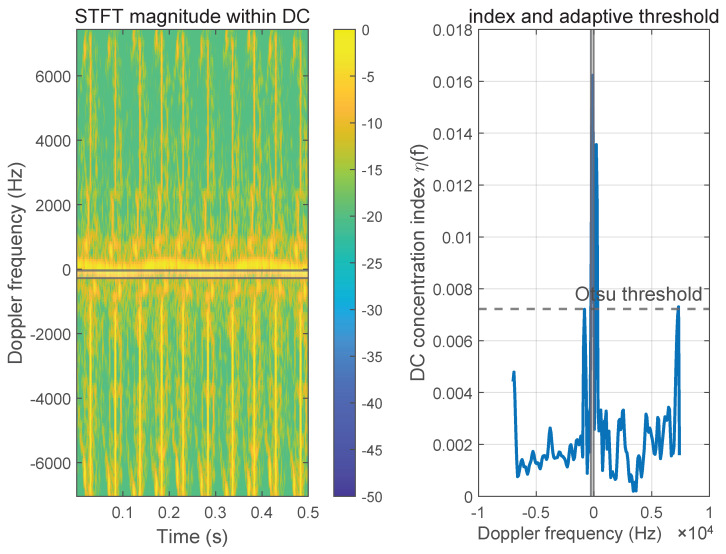
Localization of the DC-dominant overlap region.

For implementation, the DC concentration measure is first normalized by its maximum value to reduce sensitivity to absolute scaling. The DC-dominant overlap region is then localized using an Otsu-type adaptive threshold applied to the normalized concentration profile across Doppler bins. This data-driven criterion automatically separates the dominant near-zero Doppler region from surrounding frequencies by maximizing inter-class variance, yielding a contiguous interval centered at zero Doppler, identified as the DC-dominant overlap region $F_{DC}$ (Figure 3).

It should be emphasized that this adaptive thresholding step is used solely for overlap-region localization and does not participate in rotor–body allocation. The subsequent separation is governed exclusively by the CPS-guided soft weighting mechanism described in the Section 3.3.

From an implementation perspective, the DC concentration profile is evaluated on the Doppler grid defined by the CPS frequency resolution, determined by the STFT window length and cadence sampling. Increasing the frequency resolution primarily refines the smoothness of the concentration profile, while the position of its dominant near-zero region remains essentially unchanged.

It should be emphasized that $F_{DC}$ is not assumed to contain purely body-related energy; rather, it represents a band where quasi-static scattering dominates while rotor-related leakage may still be present due to finite TF resolution and low rotor speed. Hard partitioning inside $F_{DC}$ is therefore unreliable, whereas outside $F_{DC}$, rotor-induced components typically dominate and can be handled using direct assignment without soft weighting.

### 3.3. CPS-Guided Rotor Consistency Scoring

After localizing the DC-dominant overlap region $F_{DC}$, the remaining problem is not “whether” rotor components exist, but how much rotor-related leakage is present at each Doppler bin inside $F_{DC}$, where hard TF assignment is unreliable. To obtain a physically grounded cue, we exploit the fact that blade rotation induces periodic amplitude modulation in the STFT power sequence.

For each Doppler bin $f\in F_{DC}$, the STFT coefficient sequence X(t,f) is converted to a power sequence $p_{f}\left(t\right)={\left|X\left(t,f\right)\right|}^{2}$. Rotor-induced returns produce a clear periodic modulation in $p_{f}\left(t\right)$, whereas body scattering remains quasistationary without a stable modulation period. The cyclic power spectrum of $p_{f}\left(t\right)$ is then computed as:

(12) $$P_{f}\left(\alpha \right)={\left|F_{t}\left\{p_{f}\left(t\right)\right\}\right|}^{2}$$

Here α denotes the cyclic frequency. In practice, $P_{f}\left(\alpha \right)$ is evaluated over a predefined α-range covering plausible rotor rotation rates; prominent nonzero peaks indicate rotor periodicity, while body-dominant bins yield a comparatively flat CPS.

**Figure 4:**
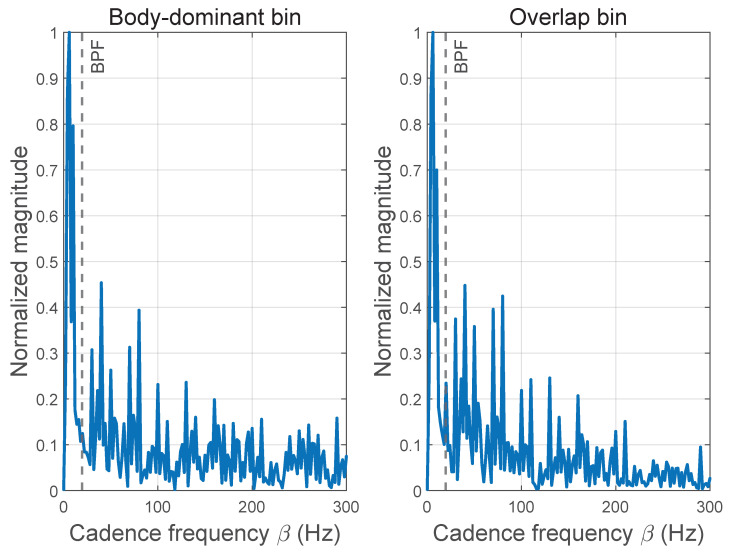
Cadence-domain signatures in different Doppler regions.

This contrast is summarized in Figure 4, which compares cadence-domain signatures extracted from different Doppler regions: rotor-dominant regions exhibit pronounced nonzero cyclic-frequency peaks, whereas DC-dominant regions show no comparable peak structure. These observations justify using CPS peak concentration as a continuous consistency cue rather than a binary detector. From an implementation standpoint, CPS evaluation is performed only for Doppler bins inside the DC-dominant overlap region $F_{DC}$, rather than across the entire TF plane. For each bin, the computation reduces to a one-dimensional Fourier transform of the STFT power sequence, and therefore introduces moderate overhead compared with iterative decomposition methods. As the processing relies on FFT-based operations over a limited frequency band, it remains amenable to offline or near-real-time radar signal processing.

Accordingly, the CPS is mapped to a rotor-consistency score. For each *f*, the score is defined as the normalized concentration of CPS energy around the dominant nonzero peak:

(13) $$s\left(f\right)=\frac{\sum_{\alpha \in A_{rot}}P_{f}\left(\alpha \right)}{\sum_{\alpha }P_{f}\left(\alpha \right)}$$

Here, $A_{rot}$ denotes a narrow neighborhood centered at the strongest CPS peak excluding α=0. Its bandwidth is determined by the CPS frequency resolution, which is set by the STFT window length and corresponds to the Doppler bin spacing in the TF domain. As long as this neighborhood covers the dominant rotor-related cyclic peak while excluding the stationary component, the resulting rotor consistency score is not sensitive to moderate variations in its width. In practice, expanding or shrinking $A_{rot}$ within one CPS resolution cell mainly affects the smoothness of the score but does not alter the relative rotor–body contrast across Doppler bins. Constructing s(f)∈[0,1], larger values indicate stronger and more coherent rotor-induced periodicity, while smaller values imply predominantly quasi-static behavior. Finally, s(f) is not used for hard rotor–body classification; instead, it serves as a bounded and physically interpretable weighting cue that will be mapped to TF-domain soft masks within $F_{DC}$ in the Section 3.4.

### 3.4. Soft Mask Construction and Signal Reconstruction

Time–frequency masking is a well-established framework for blind source separation, founded on the assumption of signal sparsity in the TF domain [27]. While binary masks are effective for strictly disjoint signals, soft weighting is preferred for overlapping components to mitigate artifacts.

With the rotor-consistency score s(f) obtained in Section 3.3, the separation inside the DC-dominant overlap band $F_{DC}$ is formulated as a soft allocation problem rather than a hard exclusion. The objective is to redistribute the overlapping energy between the body and rotor components in a controlled manner while preserving the complex STFT phase and meanwhile to avoid unnecessary modification in regions where rotor signatures are already dominant.

To this end, the score s(f)∈[0,1] is converted into a bounded rotor weight through a smooth monotonic mapping. A logistic-type mapping is adopted to avoid abrupt switching when CPS evidence is moderate and to provide a stable transition across Doppler bins:

(14) $$w_{r}\left(t\right)=\frac{1}{1+exp\{-\kappa [s(f)-\theta ]\}}$$

**Figure 5:**
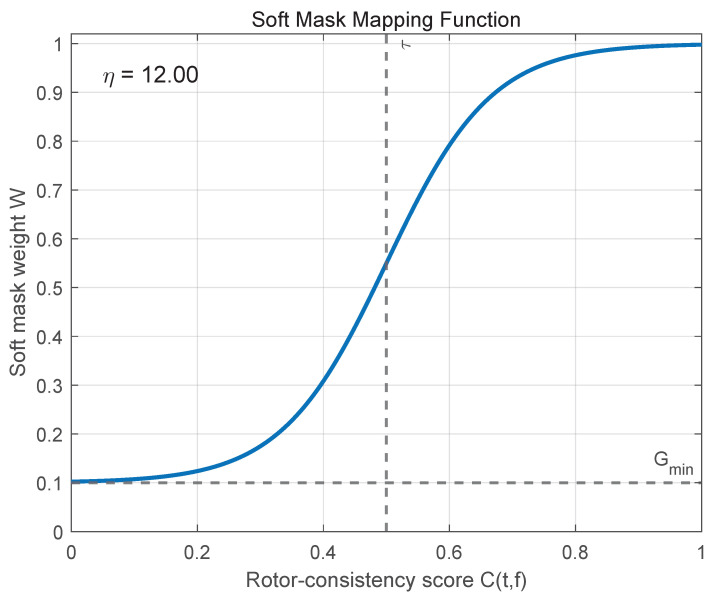
Soft Mask Mapping Function.

Conceptually, rotor–body separation can be viewed as an allocation problem on the TF plane: the body-dominated component concentrates near zero Doppler, whereas rotor returns exhibit periodically modulated ridges that intermittently enter the same region. Under such overlap, a hard partition is prone to either rotor loss (over-suppression) or body leakage (under-suppression). Motivated by this observation, we formulate a CPS-guided soft masking procedure in which the DC-dominant overlap region is first identified, rotor-related periodicity is then quantified using cyclostationary information, and soft TF-domain weighting is applied only within the ambiguity-prone region [28]. Figure 5 illustrates the CPS-guided soft mask mapping function used to assign rotor and body weights within the DC-overlap region $F_{DC}$. The rotor-consistency score s(f) is monotonically mapped to a continuous weighting function $w_{r}\left(f\right)$, yielding a center-weighted transition in which bins with stronger cyclic evidence are emphasized, while ambiguous bins near the center of $F_{DC}$ are softly shared. This formulation avoids abrupt discontinuities at the DC boundary and explains the smooth offset behavior observed in the figure.

In this mapping, $G_{\min}$ defines a lower bound on the rotor weight to prevent excessive suppression of weak but coherent rotor components and is set to 0.1 in this work. The parameter η controls the steepness of the transition from body-dominant to rotor-dominant weighting; it is selected to provide a smooth yet sufficiently discriminative transition and is kept fixed across all experiments to ensure consistent behavior. From a practical standpoint, moderate variations in $G_{\min}$ and η mainly affect the smoothness of the transition but do not alter the overall rotor–body allocation trend, since these parameters act on a bounded consistency score rather than directly enforcing hard decisions. Therefore, fixed values are adopted to balance robustness and reproducibility.

The frequency-dependent weight is then broadcast along time to form the TF-domain soft mask within $F_{DC}$:

(15) $$W_{r}\left(t,f\right)=w_{r}\left(f\right)\;\;\;\;\left(t,f\right)\in R\times F_{DC}$$

with the complementary body mask:

(16) $$W_{b}\left(t,f\right)=1-W_{r}\left(t,f\right)\;\;\;\;\left(t,f\right)\in R\times F_{DC}$$

Within $F_{DC}$, this complementary construction enables conservative soft redistribution between rotor and body components at each TF bin, while strictly preserving the total energy without introducing artificial amplification.

Outside the overlap band, the proposed framework avoids soft weighting and uses deterministic assignment. This choice follows directly from the role of $F_{DC}$: it is introduced to isolate the ambiguity-prone neighborhood around zero Doppler. For $f\notin F_{DC}$, rotor-induced micro-Doppler content typically dominates and is separable without soft redistribution, so we set:

(17) $$W_{r}\left(t,f\right)=1,W_{b}\left(t,f\right)=0\;\;\;\;\left(t,f\right)\in R\times F_{DC}$$

This “soft inside, hard outside” rule confines energy reallocation to a physically motivated region and prevents unnecessary distortion in rotor-dominant TF areas.

Let X(t,f) denote the complex STFT of the received echo. The separated TF components are obtained by applying the masks directly to the complex coefficients:

(18) $$\begin{array}{cc} X_{r}\left(t,f\right) & =W_{r}\left(t,f\right)X\left(t,f\right)\\ X_{b}\left(t,f\right) & =W_{b}\left(t,f\right)X\left(t,f\right) \end{array}$$

Operating on complex STFT coefficients preserves phase information and avoids the artifacts that may arise from magnitude-only manipulation. Finally, the time-domain rotor and body echoes are reconstructed via inverse STFT:

(19) $$\begin{array}{cc} x_{r}\left(t\right) & =ISTFT\{X_{r}\left(t,f\right)\}\\ x_{b}\left(t\right) & =ISTFT\{X_{b}\left(t,f\right)\} \end{array}$$

Recent advances in signal enhancement also highlight the importance of capturing harmonic correlations and phase patterns to achieve high-quality spectrogram reconstruction [29]. By leveraging the cyclic power as a periodic prior, our method implicitly incorporates these harmonic-aware constraints into the soft mask design.

**Figure 6:**
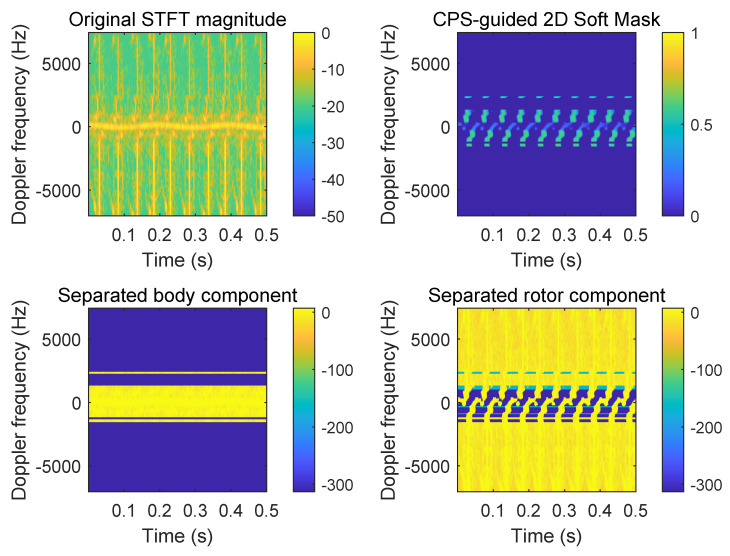
TF-Domain Separation Visual Results.

A representative TF-domain separation result is shown in Figure 6. In particular, the proposed CPS-guided soft allocation recovers rotor micro-Doppler continuity through the near-zero Doppler neighborhood, where hard DC isolation tends to remove overlapping rotor content while still suppressing residual body leakage in a controlled manner.

## 4. Experiments and Analysis

### 4.1. Simulation Setup and Experimental Configuration

To evaluate the proposed CPS-prior-guided soft mask separation under controlled and reproducible conditions, a baseline simulation is constructed using a single-rotor UAV radar echo model. The UAV is assumed to hover during the observation interval, yielding a quasi-static body return concentrated near zero Doppler, while the rotating blades generate periodic micro-Doppler modulation. This configuration represents a representative overlap scenario in which rotor leakage may partially fall into the body-dominant low-frequency band.

The simulation parameters are summarized in Table 1. The radar operates at 10 GHz with a sampling frequency of 40 kHz over an observation duration of 0.5 s. The radar-to-target range is 1000 m, and the viewing geometry is fixed at an elevation angle of 30° and an azimuth angle of 30°. The rotor speed is set to 300 rpm. For a four-blade rotor, this corresponds to a blade-passing frequency of 20 Hz, which defines the fundamental periodicity exploited in the CPS analysis and subsequent TF-domain soft allocation.

### 4.2. Visual and Quantitative Evaluation of Separation Performance

This subsection evaluates the proposed CPS-guided soft mask method under the baseline simulation in Section 4.1 using both TF-domain visualization and quantitative metrics in order to directly validate the structure-preservation and artifact-reduction objectives stated in the Introduction through comparison with hard DC isolation and data-driven decomposition baselines. To validate the proposed method against radar-relevant baselines, we consider two categories of comparison schemes. The first is hard DC isolation, a common heuristic that assigns the near-zero Doppler region to the rigid-body return. The second includes EMD and VMD, which provide data-driven decompositions for non-stationary signals without requiring an explicit scattering model. These baselines are meaningful references but are not designed to enforce the global rotational periodicity of rotor modulation and thus may suffer from mode mixing and leakage under strong DC–harmonic overlap. For reproducibility, the baseline decomposition methods are implemented with fixed and commonly used parameter settings. For EMD, the standard sifting-based algorithm is adopted, and the rotor-related component is selected as the intrinsic mode function (IMF) exhibiting dominant periodic modulation at the blade-passing frequency. For VMD, the number of modes is set to K=10 with the penalty factor 1000, and the mode corresponding to the dominant micro-Doppler band is identified as the rotor component. All baseline methods are applied to the same input signals and evaluated using identical metrics.

**Figure 7:**
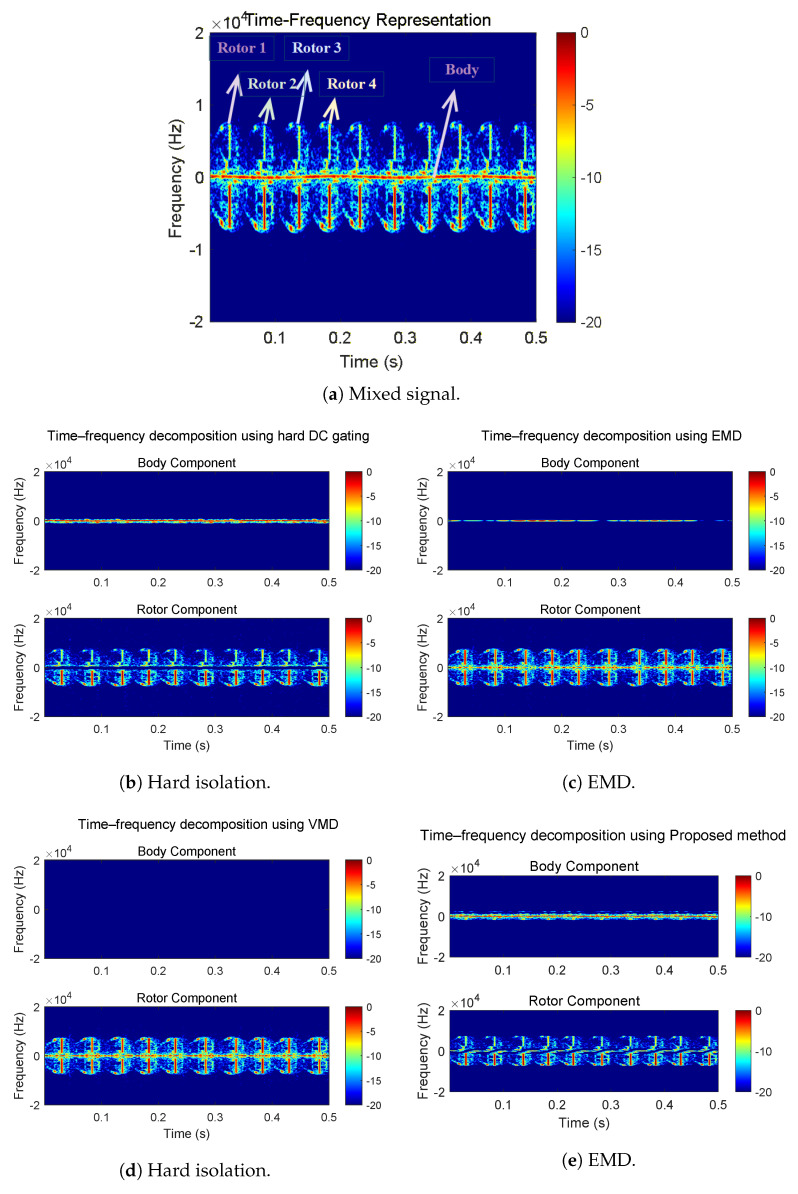
Time–frequency representations of the mixed echo and separation results.

Figure 7 compares the TF representation of the mixed echo and the rotor components extracted by different methods. In Figure 7a, a dominant near-zero Doppler ridge corresponds to quasi-static fuselage scattering, while rotor micro-Doppler trajectories exhibit periodic frequency excursions that intermittently approach the near-zero region, producing unavoidable overlap. Hard DC isolation in Figure 7b suppresses the near-zero ridge but removes the overlapped rotor content, yielding broken micro-Doppler patterns. EMD in Figure 7c shows clear mixing, with residual quasi-static energy leaking into the extracted rotor component. VMD in Figure 7d does not produce an effective decomposition in this case: under strong body dominance and shared low-frequency content, the separation tends to collapse and the dominant near-zero component is absorbed into the rotor-related mode. By contrast, the proposed method in Figure 7e preserves continuous rotor micro-Doppler trajectories while keeping the body contribution concentrated near zero, indicating a more consistent allocation inside the overlap band.

To quantify separation quality from complementary perspectives, Table 2 reports five quantitative metrics with their full names and physical interpretations. These include the Scale-Invariant Signal-to-Distortion Ratio (SI-SDR), which measures overall reconstruction fidelity; the Pearson correlation coefficient, reflecting preservation of rotor-induced temporal modulation; the rotor-band energy concentration ratio, indicating how effectively energy is confined to the expected micro-Doppler band; the time–frequency structural similarity index (TF-SSIM), characterizing the continuity and morphological integrity of micro-Doppler patterns in the TF domain; and the leakage ratio, which quantifies residual quasi-static body energy contaminating the recovered rotor component. Higher values of SI-SDR, correlation coefficient, rotor-band concentration ratio, and TF-SSIM indicate improved rotor recovery, whereas a lower leakage ratio corresponds to more effective suppression of body-to-rotor interference.

As summarized in Table 2, hard isolation reduces leakage but sacrifices overlapped rotor content; EMD and VMD suffer from pronounced misallocation and leakage under strong body dominance. In these cases, SI-SDR degrades substantially—and can even become negative—indicating that reconstruction errors outweigh the aligned rotor component due to severe mode mixing and structural distortion. By contrast, the proposed CPS-guided soft mask achieves the most favorable overall trade-off, providing higher fidelity and improved TF structure preservation with lower leakage, consistent with the visual evidence in Figure 7.

### 4.3. Robustness Analysis Under Noise Variations

Following the robustness objective outlined in the Introduction, this subsection evaluates the noise sensitivity of different separation methods by injecting additive white Gaussian noise into the complex echo, with SNR set to 5, 10, 15, 20, and 30 dB. All methods are tested under identical noise realizations to ensure a fair comparison.

**Figure 8:**
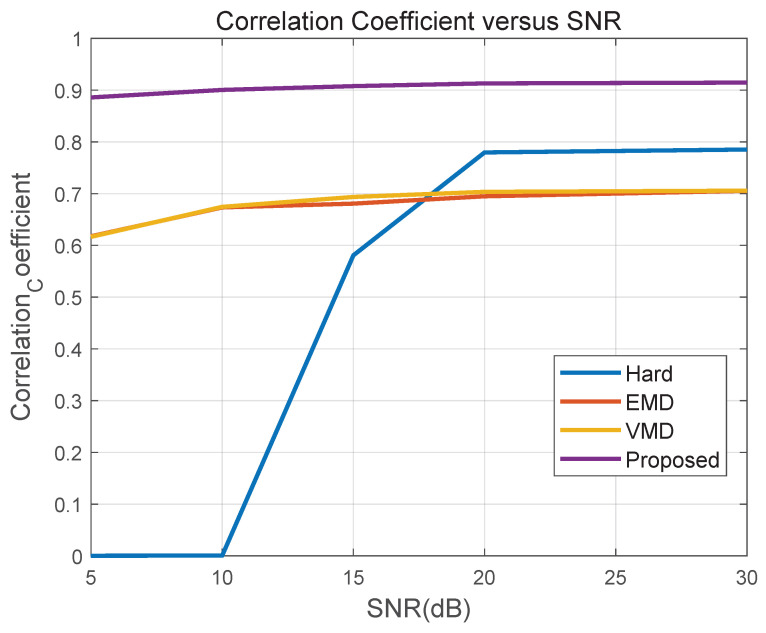
Correlation coefficient versus SNR.

Figure 8 reports the correlation between the recovered rotor component and the reference rotor signal. Correlation decreases for all methods as SNR reduces, but with distinct failure modes. Hard DC isolation can be competitive at higher SNR because rigid near-zero suppression effectively removes the dominant body return and limits noise-driven mixing. Its performance, however, drops sharply at low SNR since rotor micro-Doppler content overlapping the near-zero region is removed together with the body component, causing irrecoverable loss in the most ambiguous band. EMD and VMD degrade as SNR decreases, reflecting increased mode mixing when weak periodic modulation is submerged in noise. The proposed CPS-guided soft mask maintains the highest correlation over the full SNR range and exhibits a smoother degradation trend.

**Figure 9:**
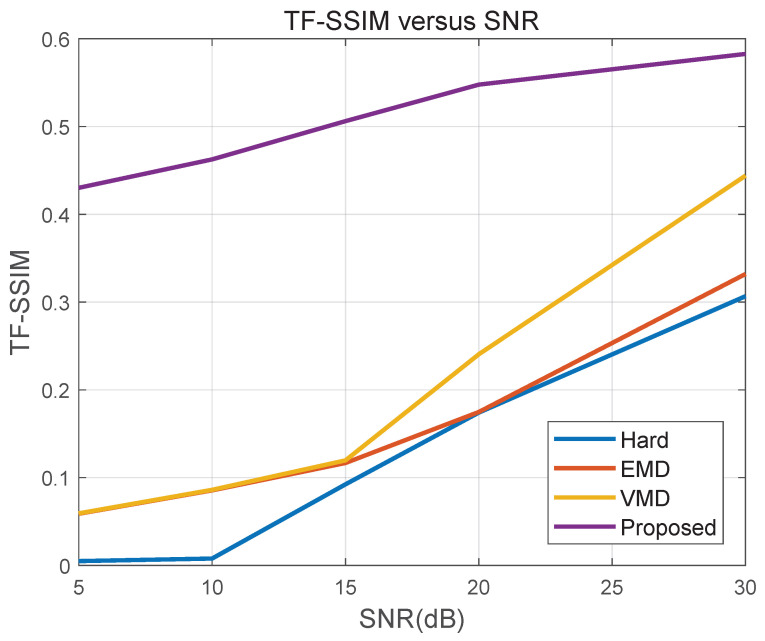
TF-SSIM versus SNR.

Figure 9 further evaluates TF-SSIM to quantify micro-Doppler structural integrity in the TF domain. Hard DC isolation yields fragmented ridges due to rigid exclusion near the overlap band, while EMD/VMD show pronounced structural distortion at low SNR. In contrast, the proposed method consistently achieves higher TF-SSIM, indicating better continuity and morphology preservation of the micro-Doppler ridge under noise.

Overall, Figure 8 and Figure 9 show that CPS-guided soft allocation degrades more gracefully with decreasing SNR. By using cyclostationary periodicity as a global statistical prior, the proposed method stabilizes energy allocation when instantaneous TF magnitude becomes unreliable, thereby preserving rotor micro-Doppler structure in noisy conditions.

### 4.4. Accuracy of Rotor Parameter Estimation

Consistent with the Introduction, this subsection evaluates rotor-speed estimation performance under varying observation conditions to examine whether preserving global rotational periodicity is sufficient for accurate and stable rotor parameter estimation in rotorcraft characterization. The simulation configurations, including radar frequency, line-of-sight angle, polarization mode, and true rotor speed, are summarized in Table 3, with the corresponding estimation results reported in Table 4. The estimation error is defined as the absolute difference between the estimated and ground-truth rotor speeds. The experimental parameters in this section are chosen to be consistent with the mathematical definitions introduced in Section 3. In particular, the Doppler bin width and CPS frequency resolution determine the low-Doppler neighborhood $V_{0}$ used in the DC concentration measure, while the CPS cadence step directly controls the frequency resolution of the rotor consistency score.

The results indicate that rotor-speed estimation is primarily governed by the preservation of global rotational periodicity rather than by fine-grained differences in rotor–body TF separation. As long as the dominant periodic component is retained, CPS-based peak localization remains stable across different sensing configurations. Accordingly, consistent rotor-speed estimates are obtained over a wide range of observation conditions.

Based on this observation, the evaluation focuses on estimation consistency rather than cross-method comparison. As shown in Table 4, the proposed framework achieves accurate and stable rotor-speed estimates without systematic degradation as sensing parameters vary.

### 4.5. Robustness Analysis of Rotor–Body Separation Under Varying Radar Conditions

In addition to rotor parameter estimation, the robustness of rotor–body separation itself is further examined under varying radar observation conditions. Six representative sensing configurations are considered, covering different line-of-sight angles, carrier frequencies, rotor rotation rates, and polarization modes, with detailed parameter settings summarized in Table 3. For each configuration, the proposed CPGSM framework is compared with hard DC partitioning, EMD, and VMD using three complementary metrics: the time–frequency structural similarity index and the leakage ratio characterizing residual body energy within the rotor-separated output.

**Figure 10:**
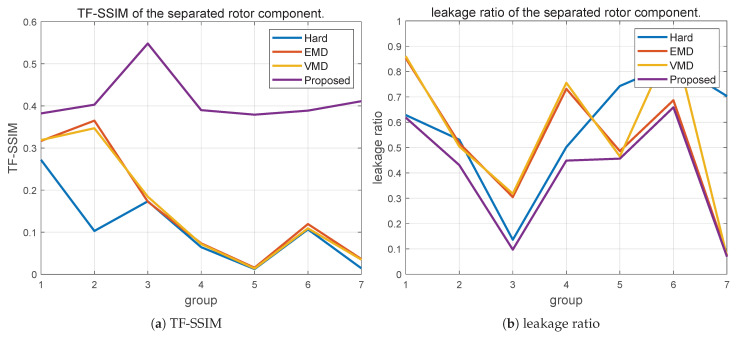
Robustness comparison of rotor–body separation.

The quantitative comparison results are summarized in Figure 10. As shown in Figure 10a, the proposed method consistently achieves higher TF-SSIM values than hard DC partitioning, EMD, and VMD across all tested configurations, indicating improved preservation of micro-Doppler continuity and the overall TF structure. Hard DC partitioning often introduces structural discontinuities near zero Doppler due to abrupt suppression, while EMD and VMD are affected by unstable decomposition and mode mixing as observation conditions vary. The leakage behavior shown in Figure 10b further supports this observation: for all six sensing configurations, the proposed method yields the lowest or near-lowest leakage ratios, reflecting more effective suppression of quasi-static body components without excessive attenuation of weak but coherent rotor returns.

Although the absolute metric values vary with radar parameters, the relative performance trends remain consistent across all configurations. Overall, these results demonstrate that the proposed CPS-guided soft masking framework provides robust and stable rotor–body separation under diverse radar sensing conditions.

From a system perspective, the effectiveness of CPS-based rotor consistency scoring depends on sufficient slow-time sampling frequency and a coherent processing interval to resolve the rotor-induced cadence. In pulse or FMCW radar systems, the slow-time sampling rate should exceed twice the maximum blade-passing frequency to avoid aliasing in the cadence domain. Insufficient sampling or overly short processing intervals may lead to broadened CPS peaks and degraded overlap-region localization. In the present simulations, the sampling rate and bandwidth are chosen to satisfy these constraints; practical deployment should ensure compliance with Nyquist criteria for both Doppler and cadence resolution.

## 5. Discussion

This work addresses a practical separation ambiguity in rotorcraft radar sensing that is driven primarily by observation geometry and line-of-sight occlusion. Under typical viewing configurations, blade returns may be intermittently shadowed by the fuselage and can collapse toward the near-zero frequency neighborhood, where they physically overlap with quasi-static body scattering. In this regime, hard DC exclusion discards meaningful rotor micro-Doppler content, while purely data-driven decompositions lack a stable physical cue to separate rotor leakage from body-dominant returns under noise.

The proposed CPGSM framework mitigates this ambiguity by combining localized overlap handling with a cyclostationary prior. Soft allocation is restricted to the DC-dominant overlap band, and the weighting is anchored to CPS-derived periodicity consistency rather than instantaneous TF magnitude. This design explains the observed advantage over hard isolation and EMD/VMD in preserving micro-Doppler continuity through the near-zero region while suppressing body leakage, especially when rotor energy is weak or partially obscured.

From a methodological perspective, this work follows a physics-guided and parameter-light design philosophy. Recent studies have explored neural-network-based strategies for micro-Doppler analysis and separation, especially under data-rich and well-annotated conditions. While such approaches can achieve strong performance through supervised learning, they typically rely on extensive training data and implicit feature representations, which may limit interpretability and robustness under unseen radar configurations. By contrast, the proposed framework explicitly exploits rotor-induced cyclostationarity as a physically interpretable prior, without data-driven model fitting.

All experiments in this study are conducted on simulated radar echoes, which allow controlled evaluation under well-defined conditions, while inevitably abstracting away certain real-world nonidealities encountered in practical radar systems.

It is worth noting, however, that the proposed framework relies primarily on cyclostationary periodicity rather than absolute amplitude information. As a result, moderate calibration errors or stationary clutter are expected to have limited impact on the CPS-based rotor consistency cue, provided that the underlying rotational periodicity is preserved. Nevertheless, systematic validation using measured radar data remains an important direction for future work.

Limitations remain. The effectiveness of the proposed framework relies on the presence of sufficiently stable rotor-induced periodicity over the coherent processing interval. When rotor speed varies rapidly within the observation window, the corresponding cyclostationary energy may spread across multiple cyclic frequencies, leading to reduced CPS peak concentration and a weaker rotor consistency cue. Similarly, blade asymmetries or intermittent occlusion can introduce irregular amplitude modulation, which degrades second-order periodicity and diminishes the contrast between rotor- and body-dominant cadence signatures. In multi-rotor scenarios, overlapping cyclic components with closely spaced rotation rates may further complicate peak attribution in the CPS domain.

In the present study, fixed mapping parameters were adopted to ensure reproducibility and to isolate the core mechanism of CPS-guided soft allocation under controlled conditions. Extending the framework to handle strong nonstationarity, complex occlusion patterns, and adaptive parameter selection—together with validation using measured radar data—constitutes an important direction for future work.

## 6. Conclusions

This paper proposed a cyclic-power-guided soft mask framework for rotor–body echo separation in rotorcraft radar signals. By explicitly localizing the DC-dominant overlap region caused by observation geometry and line-of-sight occlusion, the method avoids rigid near-zero-frequency exclusion and instead performs physically guided soft allocation based on cyclostationary evidence. As a result, weak rotor-induced micro-Doppler structures can be preserved even when they are partially embedded in body-dominant returns.

The proposed CPS-guided soft masking procedure integrates overlap localization, CPS-based rotor consistency scoring, and TF-domain soft masking in a unified framework. Simulation results demonstrate improved micro-Doppler continuity, reduced body leakage, and enhanced robustness compared with hard DC isolation and representative decomposition-based methods. Beyond parameter estimation, the proposed separation provides a foundation for component-specific motion compensation and subsequent high-resolution imaging of rotorcraft targets.

## Figures and Tables

**Table 1:** Baseline simulation parameters.

Parameter	Value
Radar operating frequency	10 GHz
Temporal sampling frequency	40 kHz
Observation duration	0.5 s
Radar viewing geometry (LOS)	Elevation = 30°, azimuth = 30°
Radar-to-target range	1000 m
Target motion state	Hovering (no translational motion)
Rotor speed	300 rpm
Blade-passing frequency (BPF)	20 Hz (for four-blade rotor at 300 rpm)

**Table 2:** Quantitative evaluation of rotor–body separation methods.

	SI-SDR	Correlation Coefficient	Rotor-Band Concentration Ratio	TF-SSIM	Leakage
Hard isolation	1.687	0.798	0.977	0.397	0.1382
EMD	−0.121	0.694	0.991	0.423	0.3040
VMD	0.193	0.706	0.992	0.531	0.3120
Proposed	6.505	0.915	0.996	0.589	0.0958

**Table 3:** Simulation settings for rotor motion parameter estimation under different operating conditions.

Case	Radar Frequency (GHz)	LOS Angles (Azimuth, Elevation)	Polarization	Observation Time (s)	Sampling Rate (Hz)	Rotor Speed (Hz)
1	6	30°, 30°	HH	0.5	40,000	5.00
2	10	30°, 30°	VV	0.5	40,000	5.00
3	10	30°, 30°	HH	0.5	40,000	5.00
4	10	50°, 30°	HH	0.5	40,000	5.00
5	10	30°, 30°	HH	0.5	40,000	5.83
6	10	30°, 30°	HH	0.5	40,000	4.17
7	10	30°, 30°	HH	0.5	40,000	4.67

**Table 4:** Rotor fundamental frequency and blade-passing frequency (BPF) estimation results.

Case	Theoretical Rotor Speed (Hz)	Estimated Fundamental Frequency (Hz)	Fundamental Frequency Error (%)	Estimated BPF (Hz)	BPF Error (%)
1	5.00	4.88	2.40	19.53	2.35
2	5.00	4.88	2.40	19.53	2.35
3	5.00	4.88	2.40	19.53	2.35
4	5.00	4.88	2.40	19.53	2.35
5	5.83	6.01	3.10	23.19	0.47
6	4.17	4.28	2.64	17.09	2.52
7	4.67	4.88	4.30	18.31	1.91

## Data Availability

The data presented in this study are available from the corresponding author upon reasonable request.
